# Macrophages in human atherosclerotic plaques in the era of single-cell and spatial transcriptomics

**DOI:** 10.1093/immhor/vlaf089

**Published:** 2026-02-17

**Authors:** Adil Ijaz, Adil Rasheed, Marco Orecchioni

**Affiliations:** Immunology Center of Georgia, Augusta University, Augusta, Georgia, USA; Immunology Center of Georgia, Augusta University, Augusta, Georgia, USA; Department of Physiology, Augusta University, Augusta, Georgia, USA; Immunology Center of Georgia, Augusta University, Augusta, Georgia, USA; Department of Pharmacology and Toxicology, Augusta University, Augusta, Georgia, USA

**Keywords:** single-cell RNA sequencing, spatial transcriptomics, macrophages, atherosclerosis, foam cell

## Abstract

Macrophages are central players of inflammation, lipid metabolism, and remodeling in atherosclerotic plaques. Historically simplified into “M1” and “M2” polarization states, their biology has been fundamentally redefined by single-cell and spatial transcriptomic technologies. Over the past decade, these approaches have identified multiple macrophage subsets within human atheromas, each driven by distinct metabolic and cytokine signatures and occupying discrete spatial niches. Human single-cell RNA sequencing (scRNA-seq), spatial transcriptomics, and multimodal omic profiling collectively demonstrate that macrophage subsets extend far beyond fixed polarization states to engage their long-recognized functions in the atheroma, including inflammation, lipid handling and repair. These findings now link macrophage identity to microenvironmental cues, vascular location, and disease stage. Importantly, these data demonstrate that these macrophages do not exist in mutually exclusive states and can transition between these subtypes in response to these aforementioned factors. Here we synthesize these advances, focusing on human data describing macrophage diversity, spatial organization, and metabolic function, and discuss how this knowledge is reshaping mechanistic models of atherosclerosis and the potential therapeutic targeting of macrophage-mediated pathology.

## Introduction

**Figure 1.**
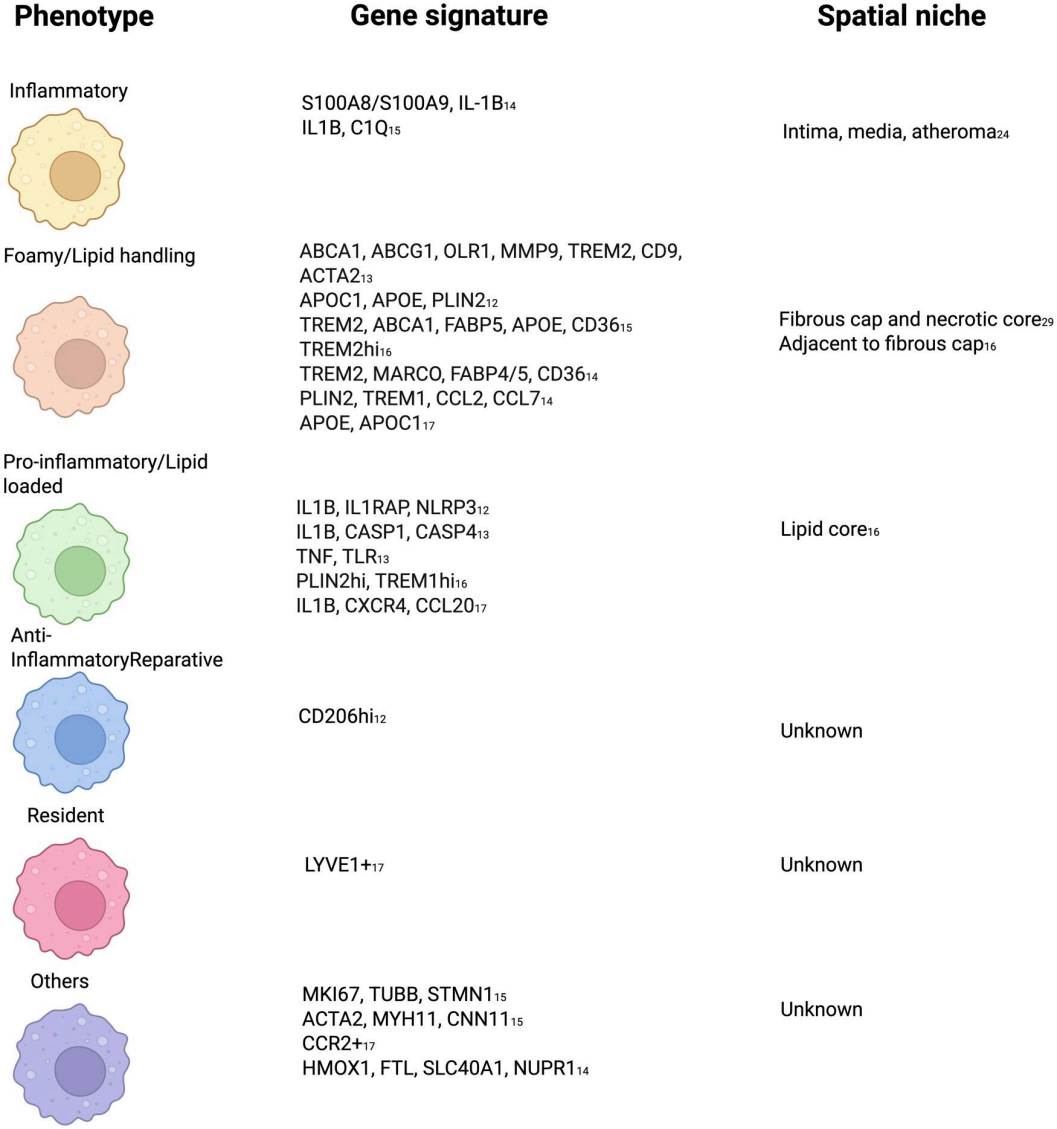
Defined macrophage phenotypes, gene signatures and spatial niche in human atherosclerotic plaques.

Atherosclerosis is a chronic inflammatory disease of large and medium-sized arteries characterized by lipid deposition and immune cell infiltration.^1^ Macrophages, both tissue resident and those replenished from circulating monocytes, are critical at every stage from early foam-cell formation to advanced stages characterized by necrotic core and plaque rupture.^1^^,^^2^ They take up modified lipoproteins, such as oxidized LDL, clear apoptotic cells, secrete cytokines, and remodel the extracellular matrix, collectively modulating lesion stability and driving clinical outcomes.^3^^,^^4^ The classical “M1/M2” paradigm, derived from *in vitro* cytokine polarization of mouse macrophages, initially provided an important framework for interpreting macrophage heterogeneity.^5^ “M1-like” cells (interferon [IFN] γ/lipopolysaccharide [LPS]-induced) were linked to pro-inflammatory functions, whereas “M2-like” macrophages (interleukin [IL] 4/13-induced) were associated with anti-inflammatory and reparative functions.^6^^,^^7^ However, this model oversimplifies the complex activation landscape within human atherosclerotic plaques, where macrophages encounter oxidized lipids, apoptotic cells, hypoxia, mechanical stress, and fluctuating cytokine gradients.^8^^,^^9^ Such environment can drive hybrid phenotypes that co-express canonical M1 and M2 markers, challenging the dichotomous classification. Initial bulk transcriptomic studies masked this underlying diversity because they average signals from mixed cell populations, as well as lack spatial information.^10^ The emergence of single-cell (sc) and spatial transcriptomics have revolutionized our appreciation for macrophage diversity in the plaque, revealing macrophages as highly heterogeneous and spatially organized populations governed by microenvironmental cues.^10^^,^^11^ In addition, multi-omics technologies, integrating chromatin accessibility, proteomics, and metabolomics, have begun linking transcriptional states to functional pathways such as lipid handling, cytokine signaling, and efferocytosis. This review discusses these findings, emphasizing discoveries in human atherosclerotic plaques that have refined concepts of macrophage diversity, spatial context, metabolism, and the translational significance of these works (Fig. 1).

## Single-cell RNA sequencing insights into macrophage diversity

The first description of immune cell heterogeneity in human atheromas using high-dimensional approaches was performed in 2019. First using mass cytometry (CyTOF), Fernandez et al. ^12^ identified two distinct macrophage subsets by surface protein expression, CD64^+^HLADR^+^CD206^hi^ and CD64^+^HLADR^+^CD206^low^, in human carotid plaques. In addition to their CyTOF data, single cell RNA sequencing (scRNA-seq) revealed 5 macrophage clusters in these plaques, showing a spectrum of macrophage phenotypes including pro-inflammatory transcriptomic profiles, inflammatory and cholesterol metabolic pathways, and a cluster consistent with a foam cell phenotype. Further addressing the contributions of these macrophage subsets to disease progression, the authors identified that symptomatic plaque macrophages were enriched in gene signatures associated with inflammation (eg *IL8*, *CXCR2*) and plaque instability (*GZMA*, *GZMK*, *GZMM*, *CCL5*). Interestingly, these macrophages expressed a decoy receptor gene, *IL1RN*, indicating potential modulation of *IL1β* signaling in these macrophages.^12^ Macrophages in asymptomatic plaques also showed a pro-inflammatory gene signature (*IL1R1*, *NLRP3*, *IL1RAP*), as well as lipid metabolism genes and elevated foam cell genes, suggesting better lipid handling capacities compared to symptomatic plaque macrophages.

Following these observations, additional studies have interrogated immune cell heterogeneity in human carotid plaques, including a combination of single-cell transcriptomics with chromatin accessibility.^13^ Work performed by Depuydt et al. identified at least 3 transcriptionally distinct macrophage populations: inflammatory, lipid-handling, and reparative.^13^ The inflammatory subset expressed *IL1β, CCL2, CXCL8*, and *NLRP3*, consistent with canonical NF-κB and inflammasome activation. Lipid-handling macrophages displayed increased expression of *TREM2*, *SPP1*, *CD9*, and *FABP5*, genes associated with cholesterol uptake and oxidative metabolism. The reparative cluster of macrophages expressed extracellular matrix and tissue remodeling genes, such as *MRC1* and *CHI3L1.*^13^ Interestingly, the lipid-handling macrophage subset shows an increased expression of *LXRα* (*NR1H3)* that is associated with an enrichment of chromatin accessibility in the region of a putative LXR_RXR binding motif (MA1149.1).^13^

Subsequent high-depth data sets^12^^,^^14–16^ have expanded this classification by integrating data across multiple vascular beds and disease stages. These analyses confirmed the recurrent presence of at least 5 macrophage states: (i) inflammatory (*IL1β*^+^, *S100A8^+^*); (ii) lipid-handling/foam (*TREM2*^+^); (iii) proinflammatory lipid-loaded (*TREM1*^+^, *PLIN2*^+^); (iv) *HMOX1*^+^ cluster, and (v) *LYVE1*^+^  *CD206*^+/−^ tissue resident macrophages (Fig. 1). Weighted gene co-expression network analysis indicated that these transcriptional subsets correlate with signaling and metabolic pathways, including NF-κB, IL-1, oxidative phosphorylation, and collagen biosynthesis.

Employing additional analyses, including trajectory inference algorithms such as RNA velocity, has deepened our appreciation for macrophage developmental transitions between these subsets. While previous analyses have described separate macrophage lipid-rich versus inflammatory subsets, a recent study has used these advanced technologies to describe the transition of *TREM2*^+^ lipid-macrophages to proinflammatory lipid-loaded macrophages (*TREM1*^+^, *PLIN2*^+^).^16^ Importantly, this work is one of the first to bridge the gap between bioinformatics and the long-standing understanding of foam cell content and the end-stage pro-inflammatory remodeling that characterizes advanced atheroma and progression to clinical events that underlie mortality. Pathway analyses from this work underscore the functional relevance of these macrophage subsets, where *TREM2*^+^ macrophages are enriched for metabolic related pathways, including Oxidative Phosphorylation and Cholesterol Metabolism, whereas the *PLIN2*^hi^/*TREM1*^hi^ population are enriched in pathways related to non-apoptotic cell death (Necroptosis, Ferroptosis), cellular stress (Response to Unfolded Protein, Mitophagy), and inflammatory pathways (NFkB Signaling Pathway, IL17 Signaling Pathway). Further interrogation of these populations reveal that these macrophages occupy spatially distinct niches within these plaques, where the *TREM2*^+^ subset is localized near the fibrous cap, with the *PLIN2*^+^  *TREM1*^+^ subset located within the lipid-rich core.^16^ While similar lipid-associated and inflammatory macrophage states have been described across murine models and human bulk transcriptomic and histological studies, direct validation of the *PLIN2^+^/TREM1^+^* population across multiple human single-cell data sets, and its transition mechanism, remain limited and warrant further research.

Comparative scRNA-seq analysis across vascular beds revealed striking context dependence in macrophage phenotypes. Slysz et al. (2023)^17^ demonstrated that carotid plaques harbor macrophages enriched for *TNF/NLRP3* signaling, while femoral lesions contain more *LYVE1^+^* macrophages expressing homeostatic and matrix-protective genes. Such site-specific profiles may reflect local hemodynamic forces, oxygenation, or lipid gradients driving the production of distinct macrophage subtypes. These results therefore underscore the importance of understanding macrophage heterogeneity in order to devise therapeutics that address local tissue insults during diseases such as atherosclerosis.^17^

Recent multi-omic integration of single-nuclei ATAC sequencing (snATAC-seq) with scRNA-seq in human plaques has identified epigenetic signatures associated with macrophage subsets where inflammatory macrophages show open chromatin regions in NF-κB and AP-1 motif dominance, whereas lipid-associated *TREM2/SPP1*^+^ macrophages exhibit a distinct chromatin repertoire (eg IRF1 and SP2/4). These data outline transcription factor hierarchies coordinating macrophage plasticity and provide deeper insights into the nuanced biology of macrophages and their response to their environment, such as inflammatory stimuli in the plaque, that influence their functional contributions to disease.^18^

## Spatial context and microenvironmental interactions

Although scRNA-seq effectively captures cells’ transcriptional heterogeneity, all information regarding the spatial distribution of these cells is lost. However, the advent of spatial transcriptomics (ST) combined with multiplex imaging to maintain anatomic context has addressed this concern and has resoundingly revealed diverse macrophage subsets occupy defined microenvironments corresponding to functional specialization and disease outcomes.

Among the most widely adopted ST approaches, 10× Genomics Visium ^19^ provides high-throughput, whole-transcriptome profiling at a near-single-cell resolution, ideal for defining transcriptional zones across plaques. NanoString GeoMx Digital Spatial Profiler (DSP) ^20^ combines multiplex RNA and protein quantification with user-defined regions of interest, allowing correlative analysis of immune, stromal, and vascular compartments. The newer CosMx Spatial Molecular Imager (SMI) ^21^ achieves true single-cell and even subcellular resolution, directly visualizing hundreds to thousands of transcripts within morphologically preserved tissue. Similarly, 10× Xenium in Situ platform^22^ offers in situ, and single-cell resolution sequencing of selected gene panels across large tissue areas with precise cellular localization, complementing transcriptomic and proteomic data.

Utilizing the Visium spatial transcriptomics platform to study human carotid plaques, Sun et al. localized rupture-site-associated macrophages.^23^ Consistent with known roles of macrophages in fibrous cap destabilization, MMP9^+^ cells were enriched in unstable/ruptured shoulders compared with stable regions, consistent with immune-activation pathways. Gastanadui et al.^24^ employed the GeoMx ST modality human coronary plaques and reported cell-specific, regionally distinct cell subsets in unstable plaques, with region-of-interest (ROI) selection based on CD68^+^ macrophage staining.^24^ Transcriptome analysis of unstable compared to stable plaques revealed enrichment of pathways involved in angiogenesis, hemostasis, immune activation, and cytokine signaling, including TNF-α and IFN-γ-mediated inflammatory cascades that may contribute to the adverse remodeling of the these unstable plaques.^24^

Recently, Campos et al. (2025)^25^ used 2 spatial transcriptomics platforms, GeoMx and CosMx, to analyze early, and advanced human atherosclerotic coronary arteries. Rather than merely identifying immune cell types, the study defined their spatial niches within atherosclerotic plaques. By integrating spatial gene expression data with immunohistochemistry images, the authors demonstrated that immune responses are spatially organized across the arterial wall. In advanced atherosclerotic lesions, macrophages are predominantly present within the plaque, localizing mainly to the intimal region, whereas T and B cells are enriched outside the plaque, particularly within arterial tertiary lymphoid organs. Furthermore, the study showed that as disease progression advances, adaptive immune cells increase in the adventitia, while macrophages become increasingly abundant within the plaque.^25^ Although Campos et al. did not resolve transcriptionally distinct macrophage subtypes, their spatial analyses demonstrate that macrophages as a class form distinct plaque-associated neighborhoods that expand with disease severity, providing an anatomical framework within which lipid-associated macrophage states described by single-cell studies are likely to operate.^25^

Overall, emerging spatial and single-cell analyses indicate that *TREM2^+^* lipid-handling macrophages accumulate adjacent to necrotic cores. The surrounding regions are enriched in *SPP1^+^* macrophages that express genes involved in extracellular matrix and remodeling,^26–29^ whereas *LYVE1^+^* macrophages populate the adventitia and perivascular zones, helping to maintain vascular integrity.^30^ Multiplex spatial proteomics corroborates this organization, revealing macrophage-endothelial interfaces enriched in regions of endothelial adhesion molecule expression such as VCAM1 and ICAM1, consistent with ongoing monocyte recruitment and vascular inflammation.^31^

In addition to transcriptomic and proteomic approaches to define the spatial heterogeneity of macrophages, recent advances in spatial metabolomics have provided an additional dimension to resolving macrophage diversity in the atherosclerotic plaque. Indeed, it is well-appreciated that metabolite availability is a physiologic stimulus for inducing switches in macrophage phenotypes.^7^^,^^32^ An example of this is a recent study that evaluated the spatial distribution of metabolites in human atherosclerotic plaque, revealing increased lactic acid in the necrotic core, potentially indicating the pro-inflammatory nature of macrophages in this region of the plaque.^32^ Together, these findings highlight macrophage spatial heterogeneity as an integrated feature of atherogenesis. Distinct subsets occupy predictable niches, including the lipid-rich core, fibrous cap, sub-intimal regions, and adventitia, corresponding to inflammation, lipid handling, plaque remodeling, and other cellular interactions. This organization underlies the simultaneous presence of destructive and reparative processes within the same lesion and emphasizes the need to interpret macrophage phenotypes within their microanatomic context. Together, these aforementioned technologies have deepened our appreciation of the molecular mechanisms that not only imprint transcriptional, proteomic, and functional heterogeneity but also these spatial gradients correlate with susceptibility with active disease processes such as rupture and thrombosis.^33^

## Functional integration and mechanistic implications

Spatial multi-omic correlation of cytokine transcripts and matrix remodeling enzymes demonstrated that *IL1B^+^/CCL2^+^* macrophages localize to areas of extracellular matrix degradation, expressing *MMP9*, *CTSL*, and *PLAUR*, thereby likely contributing to cap thinning.^29^ These cells express high levels of *NLRP3*, *CASP1*, and *GSDMD*, linking inflammasome activation to pyroptotic cell death.^34^ Consequently, these cells undergoing pyroptosis release IL-1β and additional DAMPs, further recruiting monocytes via *CCR2–CCL2* interactions and overall providing a positive feedback loop to potentiate atherogenesis.^35^  *LYVE1*^+^ macrophages display resident, tissue-homeostatic signatures with high *MRC1* and *F13A1* expression.^36^ In mice, they help maintain arterial integrity by modulating smooth-muscle collagen.^30^ Overall, a higher abundance of M2-like/adventitial macrophage features is consistent with more stable plaque phenotypes, but a specific inverse correlation with vulnerability for *LYVE1*^+^ macrophages in humans remains suggestive rather than definitive.

Integrative spatial transcriptomics and proteomics will help further map cell-cell communication networks. Recent high-resolution studies have traced macrophage differentiation trajectories using RNA velocity, indicating a gradual transition from *C1Q*/*HMOX1*^+^ to a transitional *TREM2*^hi^  *CCL18* state, and finally to a *TREM2*^hi^ population, accompanied by progressive upregulation of *SPP1*, *MMP9*, and *MMP12* transcripts.^29^ Of importance, these *C1Q/HMOX1* macrophages are subluminal, whereas the *TREM2*^hi^ population are found in shoulder regions and the vicinity of the necrotic core.^29^ These spatially layered macrophage states concentrate in the shoulder region of advanced plaques, a classically prone to rupture, and likely to orchestrate localized matrix remodeling and lipid handling.^29^ Macrophage-enriched zones near the lumen frequently co-localized with T cells and exhibited transcriptional signatures consistent with interferon-responsive signaling and adaptive immune activation. In deeper plaque layers, macrophages were located adjacent to smooth-muscle-derived cells, suggesting potential cross-talk involving matrix-remodeling and growth-factor pathways.^29^ This layered architecture strongly implicate macrophages in orchestrating inflammatory gradients across the plaque. Disruption of this equilibrium mediated by macrophages, whether through persistent metabolic stress or defective efferocytosis, could therefore tilt the balance toward inflammation and instability thereby accelerating atherogenesis.

## Translational perspectives

Studies from Zernecke et al. (2023)^36^ compared human and murine data sets, identifying conserved *TREM2*^+^ foam-cell signatures but also identifying some human-specific interferon and complement-activation pathways, reinforcing the need to interrogate human specimens in order to drive translational discoveries. Overwhelmingly, studies have demonstrated the inflammatory nature of macrophage subsets, particularly in the production of IL-1β, which perpetuates the plaque’s inflammatory milieu. This has spurred interest in neutralizing the so-called “residual inflammatory risk” in atherosclerosis. Indeed, the CANTOS clinical trial, using a monoclonal antibody against IL-1β, canakinumab, provided proof-of-concept that IL-1β blockade reduces recurrent cardiovascular events, underscoring the causal role of inflammasome-driven inflammation in human disease.^37^

Translational studies have begun linking macrophage heterogeneity to human cardiovascular risk. Integration of GWAS and chromatin accessibility data have identified putative loci within macrophage-specific super-enhancers, thereby reinforcing their causal relevance.^38^ These loci include *CD52*, *PLA2G7*, and *LIPA*. Single-cell eQTL mapping reveals that these variants modulate macrophage gene expression in cis, influencing lipid uptake and inflammatory signaling.^18^^,^^39^ As described above, TREM2 plays pivotal roles in atherosclerosis. Indeed, TREM2 risk variants, best characterized in neurodegeneration, disrupt lipid sensing/handling, microglial survival, and phagocytosis.^40^

Therapeutic targeting of macrophages has been extensively reviewed elsewhere.^41^^,^^42^ One strategy is to limit the recruitment of monocytes to the arterial wall via the CCR2/CCR5 pathway, which has shown reductions in inflammatory influx in a preclinical model.^43^^,^^44^ Another highly targeted pathway is macrophage cholesterol efflux,^45^ which can be boosted via LXR-pathway activation and PPAR-α/PPAR-γ agonists that upregulate *ABCA1*;^46^ however, this is complicated by the systemic administration of LXR ligands that induce hepatotoxicity.^47^ Therefore, direct targeting of macrophages with nanoparticles/liposomes have been investigated, such as engineered with macrophage-binding ligands (eg mannose/CD206) or plaque-targeting molecules (eg LyP-1, hyaluronan/CD44, HDL-mimetics), which can preferentially accumulate in plaque macrophages and deliver siRNA, anti-inflammatory drugs, or LXR agonists, can reduce lesional inflammation and enhance cholesterol efflux in preclinical models. Examples include mannose-functionalized dendrimers delivering LXR agonists to plaque macrophages with limited hepatic uptake,^48^ CD206-targeted siRNA micelles that improve siRNA delivery to primary macrophages,^49^ β-glucan–encapsulated siRNA that silences macrophage genes *in vivo,*^50^ statin-loaded reconstituted HDL (rHDL) nanoparticles that suppress plaque inflammation,^51^ and hyaluronan nanoparticles that home to plaque macrophages and reduce inflammation in mice.^52^ Finally, targeting the NLRP3 inflammasome with small-molecule inhibitors, most notably the NLRP3 inhibitor MCC950, suppresses IL-1β release and attenuates atherosclerosis in *Apoe^−/−^* mice and other preclinical models.^53^^,^^54^ Clinical translation, however, has been challenging: MCC950’s development encountered safety concerns, prompting a shift toward next-generation NLRP3 inhibitors.^55^ One such agent, the oral inhibitor dapansutrile (OLT1177), has shown acceptable safety in early trials (including heart failure) and functional benefits in cardiac ischemia-reperfusion models, supporting continued clinical exploration of NLRP3 blockade.^56^ Additional approaches are emerging, such as a dual NLRP1/NLRP3 inhibitor (ADS032) ^57^ and an antibody-based intracellular NLRP3 inhibitor (InflamAb),^58^ which expand the therapeutic toolkit now entering preclinical and early clinical testing.

## Future directions and unanswered questions

While single-cell and spatial multi-omics have revolutionized macrophage biology, several conceptual and technical challenges remain. Current data are predominantly cross-sectional. Without longitudinal resolution, it remains unclear which macrophage subsets drive lesion initiation, progression, or resolution. New advances in spatial multi-omics now allow measurement of transcriptomes, proteomes, and metabolites simultaneously. Merging these data will reveal post-transcriptional regulation and metabolic flux underlying the dynamic nature of macrophages in plaques. High-plex imaging mass cytometry and spatial lipidomics can help quantify metabolic processes, such as lipid catabolism and efferocytosis, directly within intact lesions. Single-cell perturb-seq and CRISPR screening are beginning to test the functional significance of macrophage regulatory gene networks.^59^ Better understanding macrophage responses and exploiting this biology will serve to overcome a major challenge in cardiovascular medicine, which is therapeutic selectivity. Global suppression of inflammation risks impairing homeostatic immunosurveillance, while targeted modulation of pathogenic macrophage subsets may offer a more effective approach. Identifying surface markers or ligand-receptor axes, such as the newly proposed olfactory receptors,^60^^,^^61^ specific to proinflammatory macrophage states, could enable precision immunomodulation. In the absence of modifiable systems, studying human cohorts entails limitations that warrant consideration. This includes the reliance on bioinformatic pipelines to extrapolate biological pathways/significance and requires formal testing to validate pursuing these perturbations in future prospective clinical trials. Furthermore, as many of these approaches reflect recent advances in these technologies, further replication is needed by independent groups and clinical cohorts. Nevertheless, these directions aim to move beyond description toward manipulation, defining how macrophage phenotypes can be therapeutically modified to promote plaque regression and stability and offer new therapeutic strategies to combat cardiovascular disease in patient cohorts.

## Conclusions

The last decade has fundamentally reshaped our view of macrophages in human atherosclerosis. New advanced technologies reveal these cells as existing along a multidimensional continuum spanning inflammatory, lipid-handling, reparative, and proliferative states. Far from their initially described role as phagocytes, macrophages dynamically integrate metabolic and microenvironmental cues to coordinate inflammation, repair, and remodeling. Understanding how these different subsets are spatially organized within the plaque, how they evolve over time, and how they can be manipulated therapeutically represents the next frontier in treating cardiovascular disease. The integration of multi-omic profiling with genetic, metabolic, and imaging approaches promises to connect descriptive cell-state maps to causal mechanisms. Ultimately, unraveling human macrophage diversity will enable the rational design of therapies that modulate, rather than suppress, innate immunity, transforming macrophages from a marker of disease to a target for cardiovascular health.

## Data Availability

There are no new data associated with this article.
